# Insufficiency fracture of the supra-acetabulum that required differentiation from a pathological fracture secondary to a malignant bone tumor: a case report

**DOI:** 10.1186/s13256-022-03642-x

**Published:** 2022-11-16

**Authors:** Sei Morinaga, Norio Yamamoto, Katsuhiro Hayashi, Akihiko Takeuchi, Shinji Miwa, Kentaro Igarashi, Hirotaka Yonezawa, Yohei Asano, Shiro Saito, Hiroyuki Tsuchiya

**Affiliations:** Department of Orthopedic Surgery, Graduate School of Medical Sciences, Kanazawa University, 13-1 Takara-Machi, Kanazawa, Ishikawa 920-8640 Japan

**Keywords:** Supra-acetabulum, Insufficiency fracture, Malignant bone tumor

## Abstract

**Background:**

The supra-acetabulum is a common site for malignant bone tumors, which can be difficult to differentiate from insufficiency fractures. We report a rare case of a stress fracture of the supra-acetabulum that required differentiation from a malignant bone tumor.

**Case presentation:**

A 74-year-old Japanese man presented to the hospital because of right hip joint pain. X-rays showed no obvious abnormalities. Magnetic resonance imaging showed an abnormality in the right supra-acetabulum, and he was referred to our department. A linear, low-signal region and its surrounding equal signal region were observed at the same site in the T1-weighted image, and a linear low-signal region and high signal region were observed in the surrounding area in the T2-weighted image. On the contrast-enhanced magnetic resonance imaging, the lesion was still unclear and the whole area was gradually enhanced. A computed tomography-guided needle biopsy was performed, but no tumor cells were observed, therefore the lesion was presumed to be a fracture healing. The bone density was 66% for the lumbar spine (young adult mean, L2–4), and blood biochemistry showed an increase in alkaline phosphatase and total type I procollagen N-terminal propeptide.

**Conclusion:**

This case was diagnosed as an insufficiency fracture of the supra-acetabulum in a male patient with primary osteoporosis by biopsy specimen. Initially, a pathological fracture associated with a malignant lesion was considered. On magnetic resonance imaging, the boundary around the fracture line was unclear and a signal change that was gradually enhanced by gadolinium was observed. This is likely to be bone marrow edema associated with the stress fracture, and we believe this to be a useful finding that may help in differentiating a stress fracture from a pathological fracture secondary to a malignant lesion.

## Background

Insufficiency fractures (IFs) are most often seen in elderly women with postmenopausal osteoporosis [1]. Also, pelvic IFs often occur in the sacrum and pubic bone, but rarely occur in the supra-acetabulum [2]. Supra-acetabular fractures due to bony insufficiency in men are not common [3].

In addition, supra-acetabulum is more commonly the site of primary malignant bone tumors and bone metastases from other malignant tumors [4]. Sakamoto *et al.* [1] described that a biopsy for the supra-acetabular lesion was needed for the purpose of diagnosis and to rule out a neoplastic lesion. We present a case of supra-acetabular IF that was difficult to diagnose.

## Case presentation

**Fig. 1:**
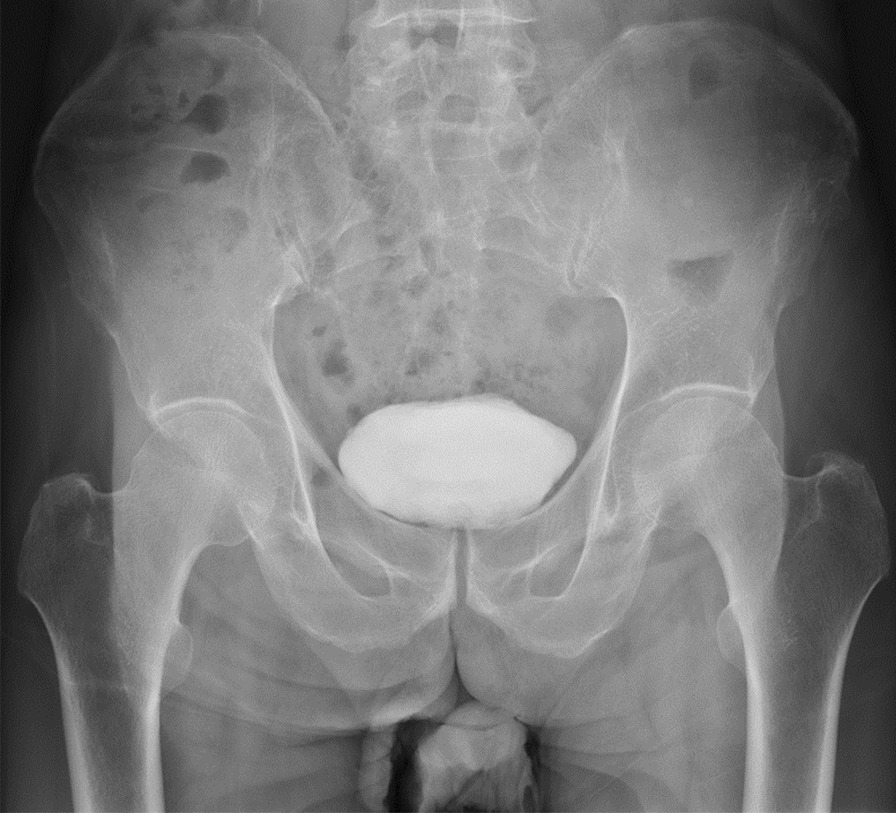
Anteroposterior pelvis radiographs taken at initial patient presentation showing no obvious abnormalities

**Fig. 2:**
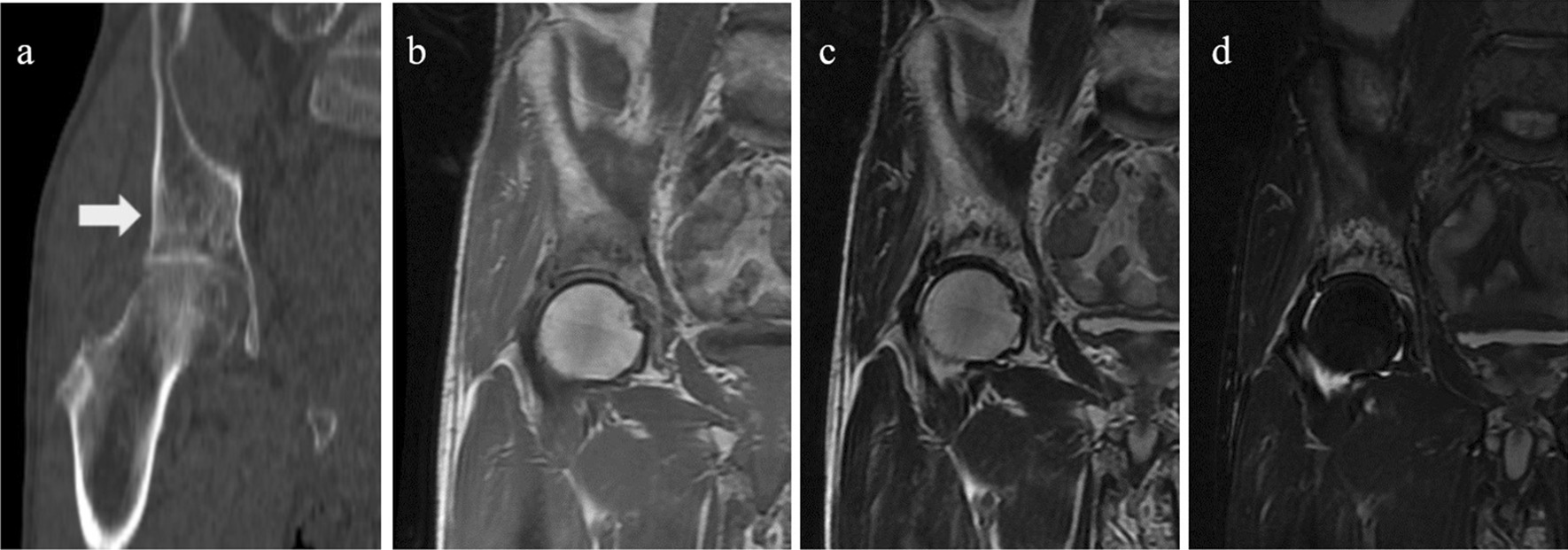
**a** Computed tomography scan showed evidence of sclerosis with a periosteal reaction in right supra-acetabulum. **b** Magnetic resonance imaging with T1 weighting showed a linear low signal and an equivalent signal surrounding it at the right supra-acetabulum. **c** The linear low signal and high signal surrounding it were seen on the T2-weighted image. **d** The lesion was not suppressed by short TI inversion recovery. The arrow show the site of the lesion

**Fig. 3:**
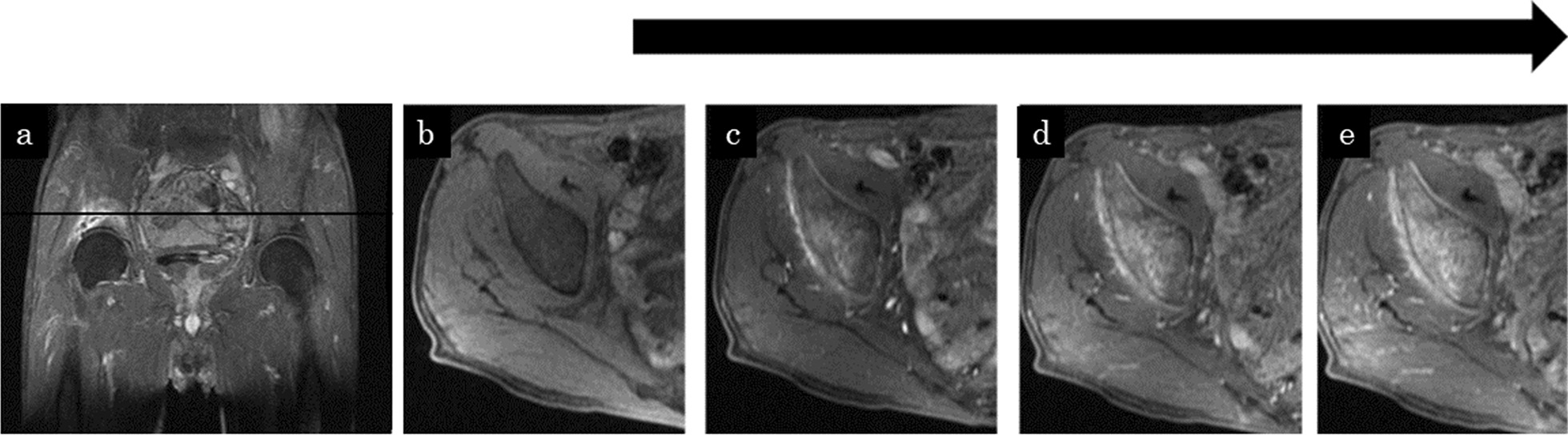
**a** Contrast-enhanced MRI, coronal plane. Black line indicates the level of the axial plane (**b**–**e**). **b** Pre-contrast-enhanced MRI, axial plane. **c**–**e** Contrast-enhanced MRI, axial plane. Black arrow indicates time passing after administration of contrast medium. They showed the lesion gradually enhanced

A 74-year-old Japanese man presented to the previous hospital complaining of right hip pain. There was no history of trauma. His history included appendicitis and hypertension. X-rays showed no obvious abnormalities (Fig. 1), so further examinations were performed. The computed tomography (CT) scan showed evidence of sclerosis with a periosteal reaction in the right supra-acetabulum (Fig. 2). Magnetic resonance imaging (MRI) with T1 weighting showed a linear low signal and an equivalent signal surrounding it at the right supra-acetabulum, and the linear low signal and high signal surrounding it were seen on the T2-weighted image. The lesion was not suppressed by short TI inversion recovery. Contrast-enhanced MRI showed the lesion gradually enhanced (Fig. 3). A malignant bone tumor at right supra-acetabulum was suspected by CT and MRI, and he was referred to our department. Blood testing was carried out for differential diagnosis considering bone metastasis from carcinoma or secondary osteoporosis. There were no inflammatory findings in the blood results, nor were there any abnormalities in his electrolytes, thyroid hormones, or tumor markers. Moreover, nuclear examinations were performed to distinguish between benign or malignant and to check whether there were other bone lesions. A bone scintigram showed accumulation in the right supra-acetabulum. Positron emission tomography showed accumulation of standardized uptake value (SUV)max3.5 in the right supra-acetabulum.

**Fig. 4:**
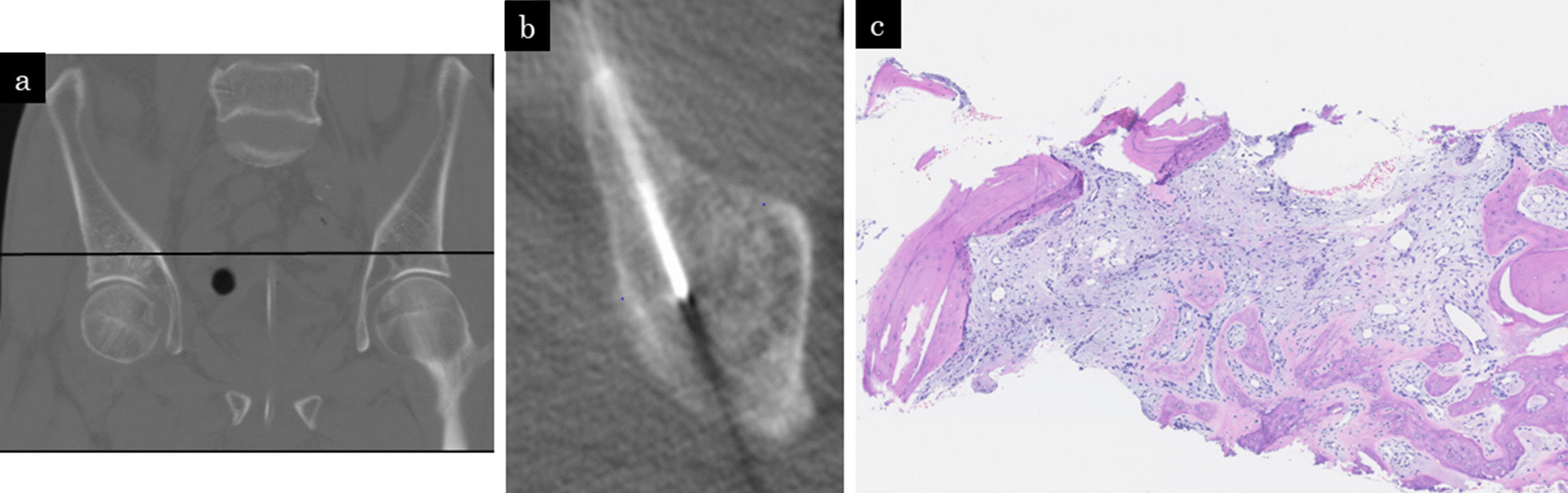
**a** Computed tomography, coronal plane. Black line indicates the level of the axial plane **b**. **b** CT-guided needle biopsy was done at the right supra-acetabulum. **c** The histological result (hematoxylin and eosin stain) showed no tumor cells, but there was formation of osteoids and infiltration of inflammatory cells, indicating the healing process after a fracture

The differential diagnoses considered were a primary malignant bone tumor such as osteosarcoma, a right supra-acetabular metastasis, and a right supra-acetabular IF. A bone biopsy was performed for diagnosis. The CT-guided needle biopsy showed no tumor cells, but there was formation of osteoids and infiltration of inflammatory cells, indicating the healing process after a fracture (Fig. 4). The pathology confirmed an IF of the right supra-acetabulum.

We performed some follow-up imaging to examine the patient for osteoporosis. The L2–L4% young adult mean (YAM) was 66% (normal value ≥ 80%), which suggested osteoporosis. Blood results included alkaline phosphatase (ALP) level at 558 IU/L (normal value 50–350), and the total type I procollagen N-terminal propeptide (totalP1NP) was 115 µg/L (normal value 18.1–74.1). These results are compatible to a healing fracture.

**Fig. 5:**
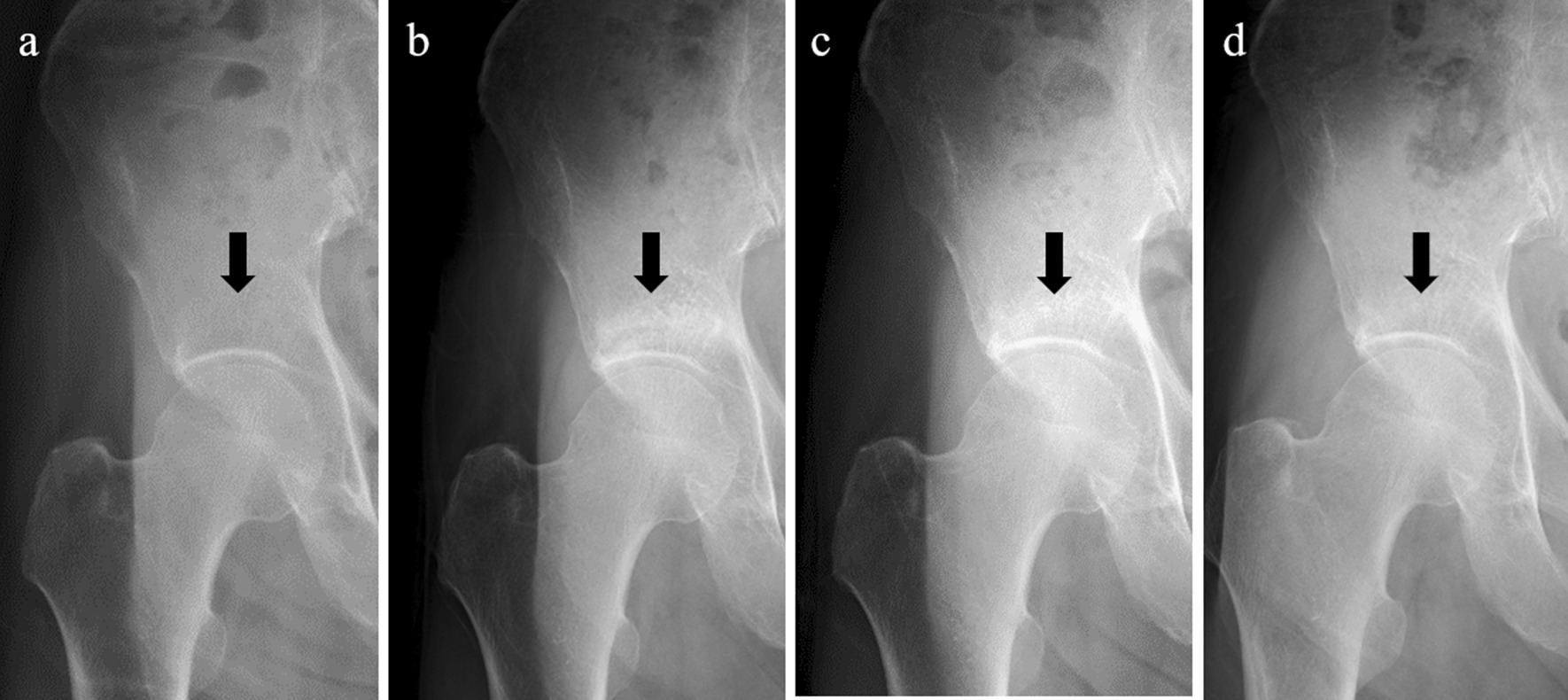
**a** X-rays at first visit showed no obvious abnormalities. **b** X-ray at 4 months after the first visit sowed sclerosis of the right supra-acetabulum. **c** X-ray at 1 year after the first visit. **d** X-ray at 2 years after the first visit showed the shadow of the sclerosis faded. The arrows show the site of the lesion

The patient was started on oral bisphosphonate once a month from 2 months after the first visit. Four months after the first visit, he no longer complained of hip pain and sclerosis could be seen on images of the right supra-acetabulum (Fig. 5). X-ray at 2 years after the first visit showed the shadow of the sclerosis faded.

No adverse and unanticipated event occurred.

## Discussion

IFs are caused by normal stresses for bone with weakened mechanical strength [5]. They commonly occur in the spine, pelvis, and lower extremities. In the pelvis, they are produced in the pubis, ilium, and sacrum [6]. Supra-acetabular IFs are rare, and this case of an IF of the supra-acetabulum required differentiation from a pathological fracture due to a malignant bone tumor.

Most IFs occur in older women with postmenopausal osteoporosis. Rheumatoid arthritis, radiation therapy, and steroid therapy are also risk factors [7]. Osteoporosis affects women four times more frequently than men [8, 9], and it is said that osteoporosis in males is more often secondary osteoporosis [10, 11]. In this case, there were no electrolyte or hormonal abnormalities, and it was suspected that the less common male primary osteoporosis was the underlying cause of the fracture.

Supra-acetabular IFs were firstly described by Cooper *et al.* [12]. There are some reports that it was difficult to distinguish from malignant bone tumors by X-ray or MRI [1, 13]. On contrast-enhanced MRI, malignant bone tumors are enhanced early [14, 15]. In this case, there were some MRI findings: fracture line curvilinear superior acetabulum; low signal intensity on T1- and T2-weighted images; significant bone marrow edema; the enhancement increased only gradually; the unclear border of the contrasted edge; and absence of associated soft tissue masses. These findings may be features for the differentiation of stress fractures from malignant bone tumors.

## Conclusion

This case of an IF of the supra-acetabulum required differentiation from a pathological fracture due to a malignant bone tumor. We propose that the signal change that was gradually lightly enhanced on contrast-enhanced MRI may be useful in differentiating IF from pathological fractures secondary to malignant bone tumors.

## Data Availability

Medical imaging data will not be shared, because it is not fully anonymous.

## Abbreviations

IF: Insufficiency fracture
MRI: Magnetic resonance imaging
CT: Computed tomography
YAM: Young adult mean
SUV: Standardized uptake value
ALP: Alkaline phosphatase
totalP1NP: Total type I procollagen N-terminal propeptide
